# Decanalization mediating gene-environment interactions in schizophrenia and other psychiatric disorders with neurodevelopmental etiology

**DOI:** 10.3389/fnbeh.2013.00157

**Published:** 2013-11-13

**Authors:** Emma L. Burrows, Anthony J. Hannan

**Affiliations:** ^1^Florey Institute of Neuroscience and Mental Health, The University of Melbourne, ParkvilleVIC, Australia; ^2^Department of Anatomy and Neuroscience, The University of Melbourne, ParkvilleVIC, Australia

**Keywords:** schizophrenia, autism, brain disorders, brain development, decanalization, gene-environment interactions, environmental enrichment, animal models

## Overview

Schizophrenia provides a striking example of a disorder in which complex genetic and environmental factors combine to produce abnormal brain development and function. In order to fully understand the disorder, and develop more effective and targeted treatments, more accurate and sophisticated animal models are required, which incorporate genetic and environmental variables and their associated gene-environment interactions. We discuss key considerations in modeling gene-environment interactions, with a focus on the recent proposal that schizophrenia involves “decanalization,” whereby “experience-expectant” brain development can have its trajectory derailed when particular genotypes (and associated cryptic genetic variants) are exposed to “unexpected” environmental conditions. This has broader implications for the modeling of schizophrenia and other brain disorders involving neurodevelopmental etiology, including autism spectrum disorders (ASDs). We propose that it is insufficient to examine animal models expressing particular genetic variants or mutations only in the single environmental context of “standard” housing conditions. The exploration of disease-associated polymorphisms and mutations under housing conditions in which environmental factors of clinical relevance are systematically manipulated will facilitate the testing of specific hypotheses associated with pathogenic gene-environment interactions and decanalized development.

## Introduction

Over the past several decades, significant advances in genetics and neuroscience have transformed our understanding of how the brain produces adaptive behavior and the ways in which normal functioning becomes disrupted in neurodevelopmental disorders, such as schizophrenia and ASD. Nevertheless, we have only begun to comprehend how particular combinations of genomes and environmental histories combine to produce a given set of clinical symptoms.

A major challenge for translating these findings to specific, effective treatments has been the heterogeneity that exists both in clinical presentation and in the genetic associations that have been uncovered. Both schizophrenia and ASD exhibit high heritability and significant research efforts have been geared toward uncovering genetic variation in a bid to explain cause. Genome-wide association studies (GWAS) have identified hundreds of common variants associated with complex diseases, however, the overall genetic risk explained by these loci remains modest (Manolio et al., 2009; Eichler et al., 2010). There is evidence for both common genetic variation and rare DNA sequence variants contributing to genetic susceptibility in both disorders (State and Levitt, 2011; Mowry and Gratten, 2013). This contribution of common and rare alleles is thought to be variable among cases, making it difficult to reliably detect an association signal among the heterogeneity and genetic noise. Adding further complexity, the genetic architecture may include epistatic (gene-gene) effects among interacting loci (Phillips, 2008). Even with sophisticated approaches to resolve gene–gene interactions acting within whole genome contexts, we are still faced with the conundrum of “missing heritability” (Manolio et al., 2009; Hannan, 2010; McGrath et al., 2011).

The problem of missing heritability may be at least partly addressed by improving the discovery rate of genetic variants via statistically well-powered cohorts of individuals that are better characterized for disease phenotypes, genetic background, and environmental exposure (Manolio et al., 2009; Eichler et al., 2010). This approach is dependent on the idea that a combination of common variants of small effect, rare variants of large effect and environmental factors will lead to disease (Manolio et al., 2009; Eichler et al., 2010; Gibson, 2011). A focus on genetic risk factors alone has limited heuristic value due to the interdependent interactions between genetic and environmental factors that play key roles in pathogenesis. An alternative view suggests that gene-environment (G × E) interactions can account for a substantial proportion of disease risk (Svrakic et al., 2013). There is strong evidence that G × E interactions are ubiquitous, accounting for the greater part of phenotypic variation seen across genotypes (Grishkevich and Yanai, 2013). Research utilizing model organisms has identified that not all genes are equally likely to exhibit G × E interactions; promoter architecture, expression level, regulatory complexity, and essentiality correlate with the differential regulation of a gene by the environment (Grishkevich et al., 2012). In fact, genes that exhibit G × E interactions may confer evolutionary advantage in that they facilitate phenotypic plasticity and provide an organism with the flexibility to adjust its phenotype with respect to the specific environmental conditions experienced (Via et al., 1995; Lande, 2009). This plasticity could represent a substantial advantage in unpredictable environments but could also, when combined with genetic susceptibility, underlie disruptions in normal brain function.

## Decanalization as a developmental manifestation of gene-environment interactions

Humans are far from being at genetic equilibrium, owing to marked changes in population size, admixture, and environment in the past few thousand years. Principle among these environmental changes are dietary shifts, modern hygiene, altered pathogen exposure, urban living, stress, and departure from natural circadian rhythm through artificial lighting. These, and other, environmental perturbations may alter the genetic contributions to phenotype by revealing cryptic genetic variation, especially among individuals with existing genetic vulnerability (Gibson, 2009; McGrath et al., 2011).

The capacity of a population to produce the same phenotype, regardless of variability in environment or genotype has been termed canalization (Waddington, 1942). Canalization is proposed to have arisen from millions of generations of stabilizing genetic selection, thus ensuring that crucial mammalian physiological mechanisms, for example, glucose metabolism, immune function, and cognitive performance remain at an optimum (Gibson, 2009). When an organism moves out of the adaptive niche, the capacity to buffer the developmental trajectory can be compromised. Decanalization, a particular class of gene-environment interaction, has been proposed as a conceptual framework for understanding how complex genetic disorders, including schizophrenia, arise (Gibson, 2009; McGrath et al., 2011). Decanalization allows cryptic genetic variation to be expressed. These mutations or polymorphisms generally don't contribute to the normal range of phenotypes observed in a population, but are thought to have a role in modifying a phenotype that arises after environmental change or the introduction of novel alleles (Gibson and Dworkin, 2004).

Human brain development may be particularly susceptible to decanalization due to rapid evolution of brain structure and function in the relatively recent *Homo sapiens* lineage (McGrath et al., 2011). Both innate genetic information and experience-dependent neural activity play critical roles in brain development. The “experience expectant” brain extracts information from the environment and uses this information to dynamically shape and refine synaptic connections. This has been clearly demonstrated by pioneering experiments showing the fundamental role of sensory input during critical periods of development for appropriate formation of connections in the visual cortex (Hubel and Wiesel, 1970). Indeed, brain development is characterized by a series of sequential gene-environment interactions; critical periods of exposure to the environment are necessary for the appropriate cortical connections to form and genetically influenced traits to be expressed. There is a consensus of evidence to show that environmental stress specifically during these critical periods of brain development in combination with a genetic predisposition increases the vulnerability to developing schizophrenia. Decades of epidemiological studies have highlighted the importance of environmental contributions to the development of schizophrenia. Several risk factors have been identified during pregnancy including season of birth, vitamin D deficiency, urbanicity or population density, and maternal viral infections (Cannon and Clarke, 2005; Brown, 2006; Patterson, 2007; McGrath et al., 2010). In addition, during adolescence, stress and cannabis abuse have been identified to negatively impact on an individual's risk of developing schizophrenia (van Os et al., 2002; Henquet et al., 2008; van Winkel et al., 2008). Animal models utilizing environmental manipulations that parallel the results of epidemiological studies in psychiatry have been critical to advancing our understanding of how the environment can modify brain function and thus the biology of psychiatric conditions. However, it is animal models incorporating gene–environment interactions that more accurately mimic etiologic factors and help to elucidate underlying pathogenic mechanisms (Gray and Hannan, 2007; Burrows et al., 2011; Kannan et al., 2013). Recent work has unmasked new phenotypes in mouse models expressing disease-associated gene mutations exposed to adverse environments, including toxins, maternal infection, stress, and drug exposure (Connor et al., 2012; Desbonnet et al., 2012; Haque et al., 2012; Abazyan et al., 2013; Karl and Arnold, 2013). Exposure to these adverse environments may lead to G × E interactions that divert brain development away from its normally canalized developmental trajectory (McGrath et al., 2011). Conversely, protective environmental factors (such as cognitive stimulation, physical exercise and a healthy diet) can induce neuroprotection and functional compensation (Nithianantharajah and Hannan, 2009). Environmental enrichment has been demonstrated to exert a range of beneficial effects on both wild-type laboratory rodents as well as animal models of brain disorders (Nithianantharajah and Hannan, 2006; Sale et al., 2009). Recent evidence suggests that an enriched environment can ameliorate behavioral traits resembling schizophrenia symptoms in genetic mouse models (McOmish et al., 2007; Harper et al., 2012), although the exact nature of the behavioral changes depends upon the gene mutations and environmental manipulations involved (Karl et al., 2007; Karl, 2013; Turner and Burne, 2013). An alternative interpretation to environmentally driven improvements is that standard housing conditions (which generally provide minimal opportunities for cognitive stimulation and physical exercise) may be actually modeling adverse environment and could interact with genetic factors to produce misleading phenotypes of reduced translational potential. Consistent with this concept, the endophenotypes modeling schizophrenia in phospholipase C-β 1 (PLC-β 1) knockout mice (McOmish et al., 2007) were most pronounced under standard housing (i.e., conditions of increased sensorimotor deprivation), consistent with the null mutation leading to decanalized brain maturation within this environmental context. Specific behavioral abnormalities were found to be absent when these PLC-β 1 knockout mice were raised under enriched conditions.

This conceptual framework also has implications for the way in which preclinical trials in animal models are designed and implemented. We have recently proposed that environmentally enriched conditions could be used as a “secondary screen” for those preclinical studies initially conducted under standard housing conditions (Burrows et al., 2011).

## New directions in modeling G × E interactions in neuropsychiatric disorders

An additional implication of this theoretical framework is that brain development may be canalized in appropriately stimulating enriched environments, but may become decanalized under conditions of sensorimotor deprivation or environmental stress. Thus, the analysis of animal models in a single “standard” housing environment has limited heuristic value (Richter et al., 2010). Animal models exploring multiple genetic and environmental forces that modify the development of pathological traits may inform mechanisms mediating decanalization and associated gene-environment interactions. Improving the validity and accuracy of animal models, including G × E interactions of clinical relevance, will increase the capacity for translation and development of new treatments (Burrows and Hannan, 2013).

The potential importance of decanalization as a mechanism mediating G × E interactions in schizophrenia is supported both by epidemiological evidence and animal models (McGrath et al., 2011). However, it would be expected that decanalization also contributes to the etiology of other complex polygenic disorders involving neurodevelopmental abnormalities.

ASDs involve abnormal brain development and contributions from both environmental and genetic factors. Furthermore, there is evidence of overlapping genetic etiologies amongst neurodevelopmental disorders such as schizophrenia and ASD (Cristino et al., 2013; Smoller et al., 2013). It is clear that the majority of ASD cases (with the exception of monogenic disorders such as fragile X syndrome) result from complex and heterogeneous genetic etiologies (Geschwind, 2011). A variety of genetic mutations and polymorphisms have been implicated in the causation of ASD, however, they are insufficient to explain key clinical and epidemiological features. This suggests that early environmental exposures also contribute. Pregnancy complications, perinatal exposure to air pollutants, maternal infection and advanced paternal age among others have all been associated with increased risk of ASD (Garbett et al., 2008; Hultman et al., 2011; Lyall et al., 2012; Roberts et al., 2013). ASD are therefore another group of major neurodevelopmental disorders that could involve decanalization. ASD can be diagnosed within the first few years of postnatal life, therefore environmental exposures *in utero* or in mothers prior to conception could be particularly important in precipitating such decanalized brain development.

Applying the decanalization framework to clinical and preclinical research may help to identify clusters of neuropsychiatric disorders that emerge from shared decanalization events. This approach has been partially utilized in the human genetics field with a recent genome-wide analysis leading to the identification of risk loci with shared effects on five major psychiatric disorders (Smoller et al., 2013). If these psychiatric disorders share early decanalization events then the current approach based on the premise that diagnoses represent distinct diseases will not differentiate the signal from the noise. Many studies currently taking this reductionist view, separating complex genetic factors from environmental exposure may in fact be missing the primary cause of these complex disorders.

A major limitation in applying this framework is the feasibility of incorporating the staggering complexity that underlies psychiatric disorders into research designs. Furthermore, simple models are not likely to be sufficient to unravel this complexity. There has been a realization that disease entities that appear to be a single disorder actually have separate developmental trajectories arising from distinct genetic vectors that influence the epigenetic landscape (McGrath et al., 2011). Recently, the director of the National Institute of Mental Health (NIMH, US), Tom Insel, and colleagues have proposed an alternative approach to address complexity in psychiatric disorders by developing a research classification system to reflect ongoing advances in genetics, neuroscience and cognitive science (Cuthbert and Insel, 2013). The Research Domain Criteria (RDoC) project, aims to support research that moves beyond descriptive syndromes in psychiatry, and toward a nosology informed by disease cause. For example, clinical trials might study all patients in a mood clinic rather than those meeting strict major depressive disorder criteria. There is scope to include gene-environment interactions in future research designs, which may provide further insight into biological mechanisms underlying psychiatric disorders.

## Conclusions

Understanding how evolved genetic programs and biological systems are dynamically sculpted by G × E interactions is the next frontier in the analysis of complex traits and in understanding the origin of neurodevelopmental disorders such as schizophrenia and ASD. In the past, G × E interactions driving variation in complex traits have been regarded by some as a nuisance, leading to difficulty in replicating results across cohorts and to the rejection of interesting genetic effects that are significant in specific environments but exhibit diminishing significance when averaged across constellations of differing environments. The realization that G × E interactions via decanalization may be integral to the development of major disorders such as schizophrenia will motivate research aimed at elucidating mechanisms as well as identifying and modifying key environmental risk factors. Furthermore, incorporation of G × E interactions into our models of neuropsychiatric disorders will provide us with the powerful tools to understand how the decanalized brain produces suboptimal phenotypes and to develop more effective therapeutic approaches.

## Abbreviations

ASD: autism spectrum disorder
G × E: gene environment
GWAS: Genome-wide association studies
PLC-β 1: phospholipase C-β 1
